# Control of Root Stem Cell Differentiation and Lateral Root Emergence by CLE16/17 Peptides in *Arabidopsis*

**DOI:** 10.3389/fpls.2022.869888

**Published:** 2022-04-18

**Authors:** Lihua Zhang, Yi Yang, Changqing Mu, Mingyu Liu, Takashi Ishida, Shinichiro Sawa, Yuxian Zhu, Limin Pi

**Affiliations:** ^1^State Key Laboratory of Hybrid Rice, College of Life Sciences, Wuhan University, Wuhan, China; ^2^State Key Laboratory of Hybrid Rice, Institute for Advanced Studies, Wuhan University, Wuhan, China; ^3^Graduate School of Science and Technology, Kumamoto University, Kumamoto, Japan; ^4^International Research Organization for Advanced Science and Technology (IROAST), Kumamoto University, Kumamoto, Japan

**Keywords:** CLE16/17, columella stem cell, stem cell differentiation, lateral root emergence, *Arabidopsis*

## Abstract

Secreted peptide-mediated cell-to-cell communication plays a crucial role in the development of multicellular organisms. A large number of secreted peptides have been predicated by bioinformatic approaches in plants. However, only a few of them have been functionally characterized. In this study, we show that two CLAVATA3/EMBRYO SURROUNDING REGION-RELATED (CLE) peptides CLE16/17 are required for both stem cell differentiation and lateral root (LR) emergence in *Arabidopsis*. We further demonstrate that the CLE16/17 peptides act through the CLAVATA1-ARABIDOPSIS CRINKLY4 (CLV1-ACR4) protein kinase complex in columella stem cell (CSC) differentiation, but not in LR emergence. Furthermore, we show that CLE16/17 promote LR emergence probably *via* activating the expression of *HAESA*/*HAESA-LIKE2* (*HAE*/*HSL2*) required for cell wall remodeling. Collectively, our results reveal a CLV1-ACR4-dependent and -independent dual-function of the CLE16/17 peptides in root development.

## Introduction

The growth and development of multicellular organisms highly rely on cell-to-cell communication which coordinates the behavior of cells with distinct identities in response to endogenous developmental cues or environmental stimuli. In plants, cell-to-cell signaling can occur by intercellular mobile factors, such as transcription factors, hormones, small RNAs, and peptides (Chitwood and Timmermans, 2010; Van Norman et al., 2011; Gallagher et al., 2014). Plant secreted peptides play critical roles in a variety of developmental processes. Full-length prepropeptides often undergo post-translational modifications, including proline hydroxylation, hydroxyproline arabinosylation, and tyrosine sulfation, and then proteolytic processing to generate mature peptides (Matsubayashi, 2014). Small peptides are secreted and mainly perceived as ligand molecules by a membrane-localized leucine-rich repeat receptor-like kinase (LRR-RK) of neighboring cells. The peptide-receptor protein complex transfers the information to alter the gene expression patterns in the nucleus through a signaling cassette in the cytoplasm, thus resulting in downstream cellular responses. It has been estimated that over 1,000 genes encode putative secreted peptides in *Arabidopsis* (Lease and Walker, 2006; Hanada et al., 2007). Thus far, only a few of them have been functionally characterized. The *CLE* gene family consists of 32 members in the *Arabidopsis* genome. The prepropeptides of each *CLE* gene contain a conserved CLE domain consisting of 12–14 amino acids, which is the mature peptide form generated by post-translational modifications and proteolytic processing. The founding member *CLV3* is expressed in the central zone of the shoot apical meristem and functions as a mobile signal to maintain stem cell homeostasis through repressing the expression of the homeodomain transcription factor gene *WUSCHEL* (*WUS*; Brand et al., 2000; Schoof et al., 2000). Apart from *CLV3*, a handful of other *CLE* gene members have also been demonstrated to be involved in various developmental processes or responses to environmental stress (for review see Fletcher, 2020).

Roots are important plant organs that are derived from the stem cells situated in the root apical meristem. A small group of cells with low mitotic activity, called the quiescent center (QC), are surrounded by stem cells in the root (Dolan et al., 1993). Elegant cellular experiments and genetic analyses have demonstrated that a mobile signal derived from the QC prevents the adjacent stem cells from differentiation (Van den Berg et al., 1997). In the root distal meristem, columella stem cells (CSCs) located underneath the QC undergo asymmetric cell division (ACD) to produce daughter cells. Those descendent cells contacted with the QC remain as new stem cells, while the rest away from the QC undergo differentiation into columella cells (CCs) containing starch granules (Dolan et al., 1993). The simple CSC system composed of QC, CSC, and CC has become an ideal model to study stem cell regulation. The homeodomain transcription factor gene *WUSCHEL-RELATED HOMEOBOX 5* (*WOX5*) is expressed in the QC and the protein moves into CSCs, where it directly represses the expression of *CYCLING DOF FACTOR 4* (*CDF4*), thus maintaining stem cells (Sarkar et al., 2007; Pi et al., 2015). The root stem cells are also specified by two independent transcription factor pathways, the GRAS proteins SHORT ROOT (SHR)-SCARECROW (SCR) and the AP2 proteins PLETHORA1/2 (PLT1/2; Aichinger et al., 2012). It has been shown that PLT1/2 forms a protein complex with SCR to directly activate *WOX5*, specifying root stem cell niche (Shimotohno et al., 2018). Other than transcription factors, peptides also play important roles in stem cell regulation. For example, root cap-expressed *CLE40* controls the root stem cell niche by confining the activity of *WOX5* in the QC (Stahl et al., 2009). Further studies have demonstrated that the function exerted by CLE40 is mediated by two LRR-RLKs, CLV1, and ACR4 which physically interact to form a protein complex in the cell membrane perceiving the peptide signaling (Stahl et al., 2013). Similar to *CLE40*, several other *CLE* members have also been implicated to repress the root meristem activity by analyzing the overexpression lines or effect of synthetic peptide application. However, no detectable root stem cell defects in the loss-of-function mutants have been reported for those *CLE* genes (Fiers et al., 2005; Kinoshita et al., 2007; Meng et al., 2010; Corcilius et al., 2017; Gutiérrez-Alanís et al., 2017; Racolta et al., 2018).

Lateral roots (LRs) are the main determinants of root systems architecture which are formed along with the primary roots. *Arabidopsis* LRs are initiated from pericycle founder cells in an auxin-dependent manner (Péret et al., 2009). After several rounds of formative cell divisions, the founder cells produce lateral root primordia (LRP) at stage I. Subsequently, the primordia progress through different stages and eventually break out the outer parental tissues to the rhizosphere, which is termed LR emergence (Malamy and Benfey, 1997; Stoeckle et al., 2018). During this process, in addition to hormones and transcription factors, multiple secreted peptides also play crucial roles as signaling ligands (Stoeckle et al., 2018). The INFLORESCENCE DEFICIENT IN ABSCISSION (IDA) peptide and its receptors HAE and HSL2 were initially discovered to function in cell separation during floral organ abscission (Butenko et al., 2003; Stenvik et al., 2008). Recently, the IDA-HAE/HSL2 signaling pathway has also been found to be required for LR emergence (Kumpf et al., 2013; Zhu et al., 2019). HAE/HSL2 bound by IDA activate the downstream MITOGEN-ACTIVATED PROTEIN KINASE (MPK) phosphorylation cascade to regulate cell wall remodeling genes such that the cell layers overlying the growing LRP can be separated, thereby facilitating LR emergence (Zhu et al., 2019).

Despite the important roles illustrated by a few identified peptides in plant growth and development, the biological function of a large number of putative peptides remains to be uncovered. Here, we report that two CLE peptides CLE16/17 promote columella cell differentiation in a CLV1-ACR4 protein complex-dependent manner. In addition, these two peptides facilitate LR emergence possibly *via* activating the expression of the *HAE/HSL2* receptor kinase genes.

## Materials and Methods

### Plant Materials and Growth Conditions

All plants are in the Columbia (Col-0) background, except for the enhancer trap line *J2341* in the C24 background obtained from the Nottingham Arabidopsis Stock Centre (NASC). Seeds were surface sterilized with 70% ethanol and germinated on 1/2 Murashige and Skoog (MS) agar plates containing 1% sucrose. Germinated seeds were grown vertically in the growth chamber under 16 h light/8 h dark at 22°C for 5 days for stem cell observations and 8 days for lateral root analysis.

The *cle16-cr1 cle17-cr1* double mutant was generated by crossing the single *cle16-cr1* and *cle17-cr1* mutants (Yamaguchi et al., 2017) and verified by Sanger sequencing. Other mutants and marker lines used in this study have been described previously: *clv1-20* (Hu et al., 2018), *acr4-2* (Gifford et al., 2003), *hae-1 hsl2-1* (Zhu et al., 2019), *pWOX5::erGFP* (Sarkar et al., 2007), *pSHR:SHR-GFP* (Nakajima et al., 2001), *pSCR:GFP-SCR* (Sabatini et al., 1999), and *pPLT1:erCFP/pPLT2:erCFP* (Galinha et al., 2007).

### Plasmid Construction and Plant Transformation

For the construction of the *pCLE16::3 × nlsGFP*/*pCLE17::3 × nlsGFP*/*pCLE20::3 × nlsGFP* reporters, a DNA fragment containing about 1.5 kb 5′ upstream and a DNA fragment containing about 1.5 kb 3′ downstream from the *CLE16*, *CLE17*, or *CLE20* coding region were amplified, respectively, from the Col-0 genomic DNA and cloned into the binary vector pGIIK containing *SV40-3 × nlsGFP* (De Rybel et al., 2011). For the constructs used in the complementation experiments of the *cle16 cle17* double mutant, a 3.3 kb DNA fragment of the *CLE16* genomic sequence and a 3.4 kb DNA fragment of the *CLE17* genomic sequence were amplified from Col-0 genomic DNA, respectively, and cloned into the binary vector NK43-Tn. All the constructs were transformed into plants by the agrobacterium floral dip method (Clough and Bent, 1998). All primers used for PCR amplification are listed in Supplementary Table 1.

### Microscopy

For confocal laser scanning microscopy (CLSM), roots were stained with 10 μg/ml propidium iodide (PI, Sigma P-4170) and images were taken with LAS-AF-Lite 3.3 software on Leica DM6 CS confocal microscope. To image fluorescent proteins and PI simultaneously, the sequential scanning mode was used. GFP was excited using a 488 nm laser line in conjunction with 500–545 nm collection; PI was excited using a 552 nm laser with 600–700 nm collection. Images were further processed using Leica LAS-AF-Lite software. The fluorescent protein intensity is represented by the average mean gray value. Modified pseudo-Schiff propidium iodide (mPS-PI) staining was performed as previously described (Truernit et al., 2008).

For analysis of developmental stages of lateral roots, peptide-treated or 8 days post-germination (dpg) seedlings grown vertically on agar medium were collected and cleared according to Malamy and Benfey (1997). All developmental stages of LRP and emerged lateral roots were imaged and counted with LAS V4.12 software on Leica DM2500 differential interference contrast (DIC) microscope.

### Peptide Treatment

CLE5p (RVSPGGPDPQHH) and CLE16p (RLVHTGPNPLHN) were chemically synthesized (GenScript, 95% purity). The peptides were dissolved in mili-Q water and added into the agar medium to reach a final concentration of 1 μM. For observing root stem cells, seeds were germinated on agar medium with or without 1 μM peptides. For analyzing LRs, seedlings at 6 dpg were transferred into the medium with or without CLE16p for 18 h or 2 days as indicated in figure legends (Péret et al., 2012).

### Root-Bending Assay

Agar plates with seedlings at 6 dpg grown vertically were turned 90° so that roots were gravistimulated for 18 h. The roots were collected and cleared by chloral hydrate, then the LRP at the bend were imaged and analyzed by Leica DM2500 DIC microscope.

### Quantitative Reverse Transcription PCR

Total RNA was extracted from 8 dpg roots using the FastPure Plant Total RNA Isolation Kit (Vazyme RC401). RNA was reverse transcribed using the HiScript II First Strand cDNA Synthesis Kit (Vazyme R211). Quantitative PCR was performed with the ChamQ SYBR qPCR Master Mix (Vazyme Q311) on CFX Real-Time PCR Detection System (Bio-Rad). Three biological replicates for each sample and two technical replicates per biological replicate were performed and the data were analyzed by CFX Maestro Software. Two reference genes were used for normalization (Czechowski et al., 2005). All primers used are listed in Supplementary Table 1.

### Statistical Analysis

Statistical significance of normally distributed data was tested by either Student’s *t*-test or one-way ANOVA followed by Dunnett’s multiple comparisons test as indicated in figure legends. Statistically significant differences are indicated by ^*^p < 0.05, ^**^p < 0.01, and ^***^p < 0.001.

## Results

### Expression Patterns of *CLE16/17/20* in Root

**Figure 1:**
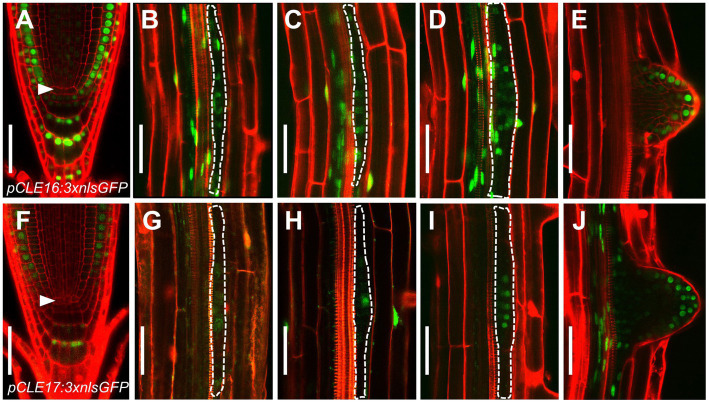
CLAVATA3/EMBRYO SURROUNDING REGION-RELATED (*CLE*) promoter activity in roots. **(A–J)** Representative expression patterns of *CLE16* **(A–E)** and *CLE17* **(F–J)** in primary root tips at 5 days post-germination (dpg; **(A,F)** early lateral root primordia (LRP) at stage I–III **(B–D,G–I)** and emerged lateral roots **(E,J)**. GFP, green; propidium iodide, red. Arrowheads indicate the quiescent center (QC) cells **(A,F)**. LRPs are outlined with dotted lines **(B–D,G–I)**. Scale bars represent 50 μm.

Our previous phylogenetic analysis using the full length of all 32 CLE proteins shows that *CLE16*, *CLE17*, and *CLE20* are clustered into a subfamily (Yamaguchi et al., 2017). As a first step to identify the function of this gene subfamily, we determined the promoter activity of each *CLE* gene in roots. To this end, we constructed three transcriptional reporters using an approximate 3 kb genomic sequence 5′ upstream plus 3′ downstream from the coding region of each *CLE* gene to drive the expression of three times green fluorescence protein with nuclear localization sequence (*pCLE:3 × nlsGFP*). Transcriptional reporter gene analysis revealed that *CLE16* is expressed in root cap, epidermis, and also weakly in CSCs, but not in the QC (Figure 1A). Additionally, *CLE16* is expressed uniformly in LR primordia from stage I to III and is gradually restricted to the outermost cell layers in the emerged LR primordia (Figures 1B–E). *CLE17* displays a similar expression pattern to *CLE16*, but with a lower strength (Figures 1F–J). The GFP signal was not detected in the root tips of *pCLE20:3 × nlsGFP* (Supplementary Figure 1). Consistent gene expression patterns of these three *CLEs* in root were revealed by published single-cell RNA sequencing (scRNA-seq) data available online (Wendrich et al., 2020; Supplementary Figure 1). As *CLE20* is not expressed in the primary root meristem, we excluded it in the subsequent studies.

Together, these gene expression data indicate that *CLE16* and *CLE17* function in root cap differentiation and lateral root formation.

### *CLE16/17* Promote Stem Cell Differentiation

**Figure 2:**
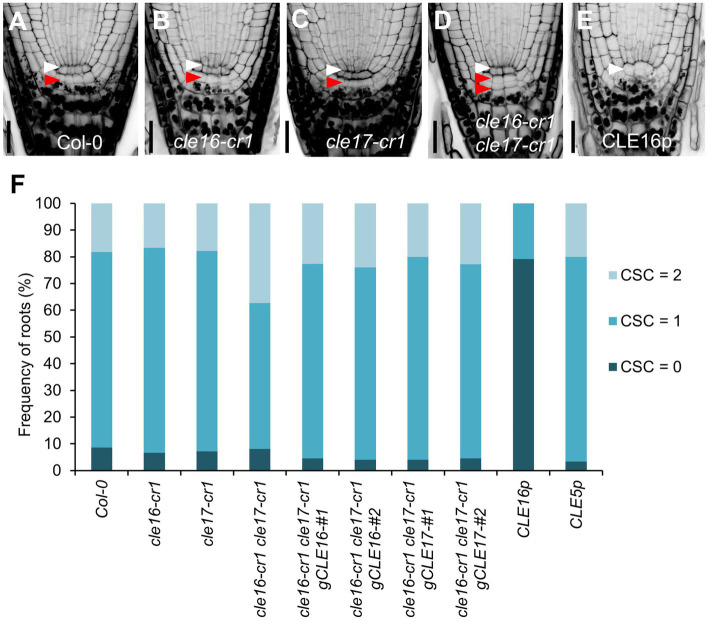
*CLE16*/*17* promote columella stem cell (CSC) differentiation. **(A–E)** Representative confocal images of the root tips at 5 dpg stained with mPS-PI in Col-0 **(A)**, *cle16-cr1* **(B)**, *cle17-cr1* **(C)**, *cle16-cr1 cle17-cr1* **(D)**, and 5 dpg Col-0 grown on agar medium with 1 μM CLE16 peptides (CLE16p). White and red arrowheads indicate the QC cells and CSCs, respectively. Scale bars represent 25 μm. **(F)** Quantification of CSC differentiation characterized by the accumulation of starch grains (gray or black dots) in the indicated genotypes. Two independent transgenic lines harboring genomic DNA fragment *gCLE16* or *gCLE17* were analyzed for the complementation test in *cle16-cr1 cle17-cr1* double mutants. Roots with differentiated CSCs (0), one layer of CSCs (1), or two layers of CSCs (2) were counted (*n* > 50 for each genotype).

To determine the biological roles of the *CLE16/17* genes in the root, we characterized the developmental phenotypes of their loss-of-function mutants. *cle16-cr1* and *cle17-cr1* are null alleles generated by CRISPR/Cas9-mediated gene editing, both of which contain a 1-bp insertion before the mature CLE peptides in the coding region (Yamaguchi et al., 2017). The primary root length and meristem size of *cle16-cr1*, *cle17-cr1*, and *cle16-cr1 cle17-cr1* are indistinguishable from wild-type (Supplementary Figure 2). Because *CLE16/17* are expressed in columella cells, we scrutinized the development of the distal meristem in mutants. No obvious phenotypes were observed for *cle16-cr1* or *cle17-cr1* single mutants (Figures 2A–C,F). While in *cle16-cr1 cle17-cr1* double mutants, we found a significant increase of the frequency of roots with two CSC layers without accumulating starch granules compared with wild-type (37.4 vs. 18.3% of Col-0; Figures 2A,D,F), indicating that the differentiation of the columella cells in double mutants are delayed. These defective phenotypes of cell differentiation in the double mutants could be fully complemented by introducing an about 3.3 kb genomic fragment of *CLE16* or *CLE17* (Figure 2F). Previous studies have shown that synthetic CLE peptides can activate the downstream signaling pathway to inhibit root growth (Fiers et al., 2005; Stahl et al., 2009; Meng et al., 2010; Racolta et al., 2018). Thus, we further assessed the function of the *CLE16/17* genes in the root by exogenous application of synthetic CLE16 peptides (CLE16p). Similar to the effects of many synthetic peptides including CLE17p (Whitford et al., 2008; Stahl et al., 2009; Racolta et al., 2018), treatment of CLE16p resulted in shorter root and reduced meristem size compared with non-treated wild-type (Supplementary Figure 2). Moreover, the application of CLE16p led to the accumulation of starch grains underneath the QC in 79.2% of wild-type roots (Figures 2E,F), indicating that CSCs have undergone differentiation. As a negative control, treatment of CLE5p did not cause any phenotype distinct from wild-type (Figure 2F; Supplementary Figure 2), indicating that the above inhibitory effects are specific to CLE16p and structurally similar CLE peptides. Together, these results suggest an important role of *CLE16/17* in stem cell regulation.

**Figure 3:**
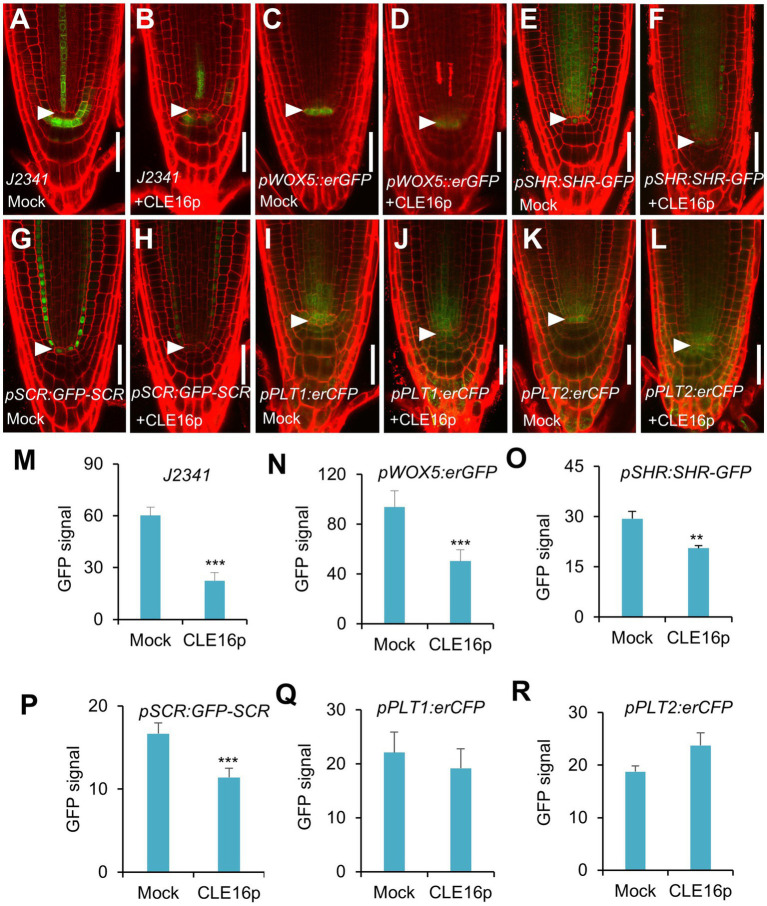
Expression analysis of stem cell identity markers in roots with no peptide (Mock) or with CLE16p treatments. **(A–L)** Confocal images showing the representative expression patterns of *J2341* in mock control **(A)** and CLE16p treatments **(B)**, *pWOX5:erGFP* in mock control **(C)** and CLE16p treatments **(D)**, *pSHR:SHR-GFP* in mock control **(E)** and CLE16p treatments **(F)**, *pSCR:GFP-SCR* in mock control **(G)** and CLE16p treatments **(H)**, *pPLT1:erCFP* in mock control **(I)** and CLE16p treatments **(J)** and *pPLT2:erCFP* in mock control **(K)**, and CLE16p treatments **(L)**. Roots were grown on agar medium supplemented with or without 1 μM CLE16p for 5 days. Arrowheads indicate the QC cells. Scale bars represent 50 μm. **(M–R)** Quantification of GFP signals in **(A–L)**. Error bars represent SD (*n* = 10 for each genotype). Student’s *t*-test, ^*^p < 0.05, ^**^p < 0.01, and ^***^p < 0.001.

To gain a better insight into the molecular function of the CLE16/17 peptides, we analyzed the effects of their treatment on the expression of stem cell identity markers in the root meristem. Consistent with the observation of the accumulation of starch grains at the position of CSC (Figure 2E), the expression of the CSC marker *J2341* is greatly reduced upon the CLE16p treatment (Figures 3A,B,M). We next asked whether QC identity is also influenced by the peptide treatment. In comparison with mock-treated control, the *pWOX5::erGFP* expression is significantly decreased in the roots treated with CLE16p, suggesting that the QC identity is impaired (Figures 3C,D,N). Previous studies have shown that the SHR-SCR and PLT1/2 modules activate WOX5 to specify the root stem cell niche (Shimotohno et al., 2018). Therefore, we tested whether the decreased *WOX5* expression is due to the attenuated activity of those upstream transcriptional regulators. Indeed, the protein levels of both SHR-GFP and GFP-SCR are remarkably decreased upon the CLE16p treatment (Figures 3E–H,O,P). By contrast, the expression levels of PLT1/2 are not significantly altered (Figures 3I–L,Q,R). We, therefore, conclude that CLE16p promotes the differentiation of the QC through repressing the *SHR-SCR-WOX5* pathway.

### Requirement of the CLV1-ACR4 Protein Complex for the CLE16/17 Signaling

**Figure 4:**
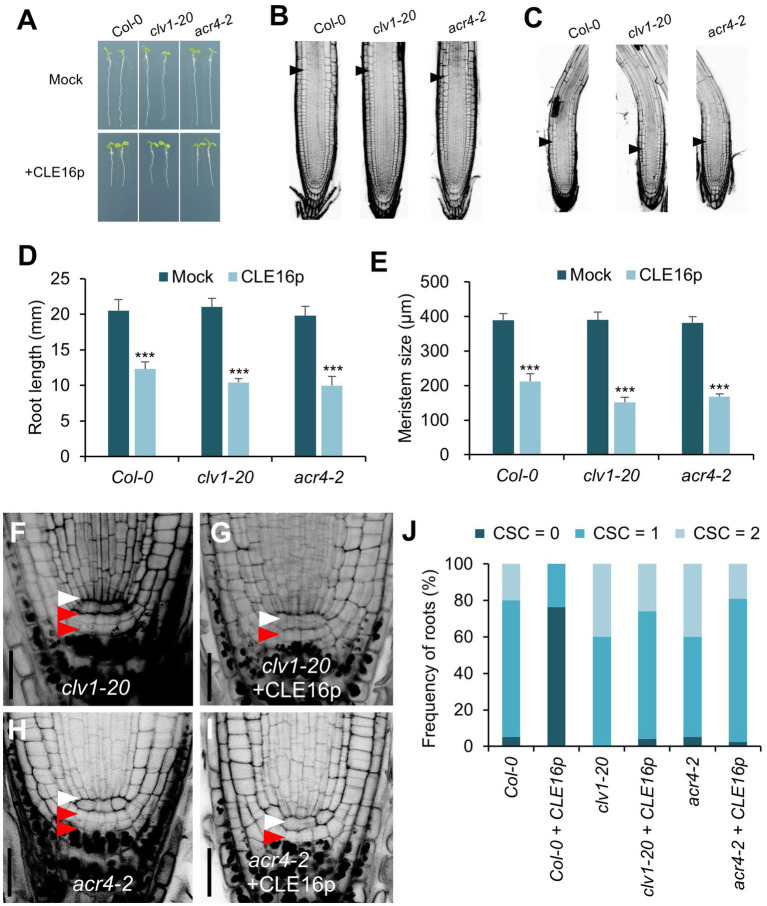
CLV1-ACR4 complex acts in the CLE16 signaling pathway to promote columella cell differentiation. **(A)** Seedlings at 5 dpg were treated without (top row) or with 1 μM CLE16p (bottom row). **(B,C)** Confocal images of roots stained with PI after mock treatment **(B)** or CLE16p treatment **(C)**. Black arrowheads indicate the junction between meristematic and elongation zones. **(D,E)** Quantification of root length **(D)** and meristem size **(E)** of the genotypes shown in **(A)** and **(B,C)**, respectively. Error bars represent SD. *n* > 20 for each genotype. Student’s *t*-test, ^*^p < 0.05, ^**^p < 0.01, and ^***^p < 0.001. **(F–I)** Representative confocal images of the root tips stained with mPS-PI after mock treatment **(F,H)** or CLE16 treatments **(G,I)**. White and red arrowheads indicate QC cells and CSCs, respectively. **(J)** Quantification of CSC differentiation of the indicated genotypes. *n* > 50 for each genotype. Roots were grown on agar medium supplemented with or without 1 μM CLE16p for 5 days **(A–J)**.

It has been shown that the CLV1-ACR4 protein complex perceives the CLE40 signal to regulate root growth (Stahl et al., 2009, 2013). Therefore, we next tested whether the same complex is involved in the perception of CLE16/17. *clv1-20* and *acr4-2* mutants are morphologically indistinguishable from wild-type in terms of root length and root meristem size (Figures 4A,B,D,E). When grown on MS agar plates containing CLE16p, the seedlings of wild-type, *clv1-20*, and *acr4-2* display sensitivity to peptide treatment as shown by the shorter roots and reduced root meristem size compared to mock-treated control (Figures 4A–E). These results suggest that the inhibitory effect of CLE16p on the proximal root meristem size and consequently root growth is independent of the CLV1-ACR4 protein complex. We next asked whether the CLV1-ACR4 complex is required for inhibiting distal root stemness regulated by CLE16/17. Consistent with previous results (Stahl et al., 2009, 2013), both *clv1-20* and *acr4-2* mutants exhibit increased frequency of roots having an extra undifferentiated CSC layer compared to wild-type (Figures 4F,H,J). In contrast to wild-type, the effect of CLE16p on promoting CSC differentiation is largely suppressed in *clv1-20* and *acr4-2* roots (Figure 4J). In summary, these results suggest that CLE16/17 promote columella cell differentiation through the CLV1-ACR4 protein kinase complex.

### CLE16/17 Regulate Lateral Root Emergence

**Figure 5:**
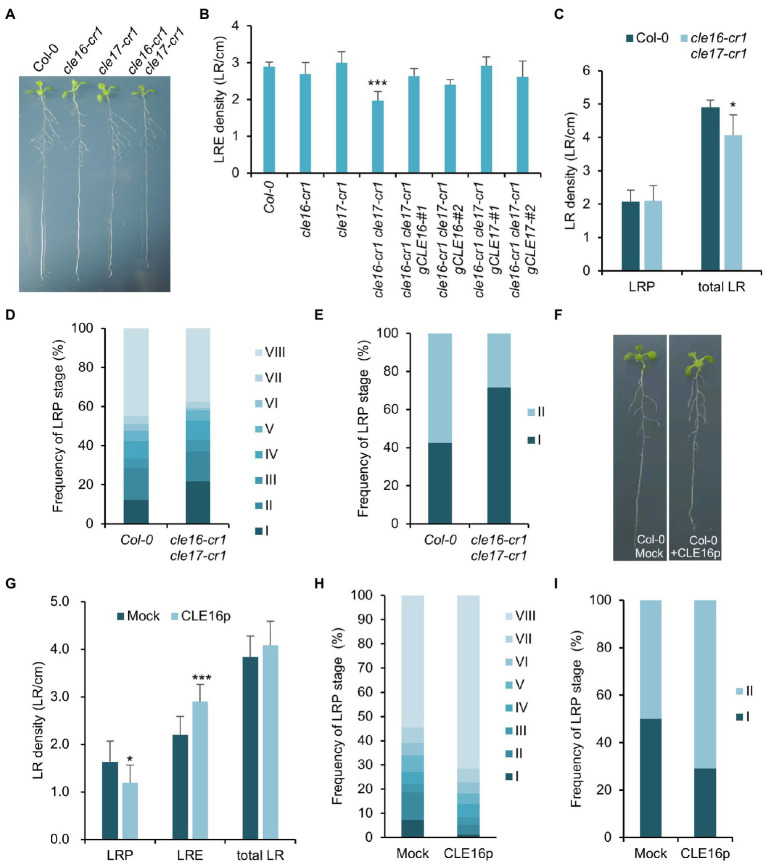
*CLE16/17* are required for lateral root emergence. **(A)** Phenotypes of the seedlings of *cle16/17* double mutants at 8 dpg. **(B)** Quantification of lateral root emergence (LRE) density in the *cle16/17* double mutants. *n* = 30 for each genotype. **(C)** LR primordia (LRP) and total LR (LRE + LRP) density of wild-type (Col-0) and *cle16/17* at 8 dpg. *n* = 35 for each genotype. **(D)** Quantification of LRP at various stages in Col-0 and *cle16/17*. Stages I–VIII are color-coded. *n* = 30 for each genotype. **(E)** Quantification of the initiated LRP at stage I or II in Col-0 and *cle16/17* after primary root bending for 18 h. *n* = 30 for each genotype. **(F)** Seedlings of Col-0 at 8 dpg after mock or 1 μM CLE16p treatment for 2 days. **(G–I)** Quantification of LRP, LRE, and total LR density in seedlings treated with or without 1 μM CLE16p. **(H)** Quantification of individual primordia stages in seedlings treated with or without CLE16p. **(I)** Quantification of the initiated LRP at stage I or II in Col-0 treated with or without CLE16p after primary root bending for 18 h. *n* = 30 for each treatment. Error bars represent SD. Student’s *t*-test, ^*^p < 0.05, ^**^p < 0.01, and ^***^p < 0.001.

The observation that *CLE16/17* are expressed in early LRP led us to investigate whether they also play a role in lateral root development. Neither *cle16-cr1* nor *cle17-cr1* single mutants show obvious defects in later root emergence (LRE) compared to wild-type (Figures 5A,B). Interestingly, in comparison with wild-type, the *cle16-cr1 cle17-cr1* double mutants show significantly less emerged lateral roots (Figures 5A,B). Notably, this defect in double mutants can be restored by introducing a genomic DNA fragment of *CLE16* or *CLE17*, indicating that mutations of *cle16-cr1* and *cle17-cr1* indeed cause the lateral root phenotypes (Figure 5B). Given that no significant difference in the LRP but significantly lower total LR density was observed in the *cle16-cr1 cle17-cr1* double mutants compared to wild-type (Figure 5C), we thereby conclude that *CLE16/17* promote lateral root emergence rather than initiation. To further reveal the regulatory role of *CLE16/17* in the lateral root emergence, we conducted a detailed analysis of all LR developmental stages in the double mutants. The proportion of the LR primordia at stage I in *cle16-cr1 cle17-cr1* double mutants was found to be remarkably increased compared to wild-type (21.9% versus 12.4% of wild-type; Figure 5D), indicating that the loss of *CLE16* and *CLE17* activities lead to the prolonged transition from stage I to II, and thus eventually less emerged LRs. It has been reported that gravitropic curvature or a transient bending by hand can induce the LR initiation in an auxin-dependent manner at the outer side of a root bend (Ditengou et al., 2008; Laskowski et al., 2008). We used this bending system to confirm the function of *CLE16/17* in LRE. In wild-type, approximately 40% of the induced LRP are at stage I, and the rest are at stage II after primary root bending for 18 h (Figure 5E; Péret et al., 2012). While in the *cle16-cr1 cle17-cr1* double mutants, the frequency of LRP at stage I is increased to 71.5% under the same treatment (Figure 5E), confirming the delayed developmental progression of LRP at stage I in the *cle16 cle17* double mutants under normal growth conditions. We next examined the effects of exogenous CLE16p treatment on lateral root development. To reduce the secondary effects on lateral root formation from inhibited primary root growth by the CLE16p application (Supplementary Figure 2), the wild-type seedlings were treated with the CLE16p peptide for a short period instead of germinating on the peptide-containing agar plate. As expected, we observed that CLE16p-treated roots are just slightly shorter than mocked-treated ones (Figure 5F). Consistent with the results of the loss-of-function *cle16 cle17* mutants, CLE16p treatment does not alter the total LR density, but conversely results in significantly less LRP and more LRE in comparison with mock treatment (Figures 5F,G). In addition, exogenous application of CLE16p substantially accelerated the developmental progression of LRP from stage I onwards as shown by a much lower percentage of early LRP stages, particularly for stage I compared to the mock control (Figure 5H). This promotion effect of LRP by CLE16p was also confirmed in an independent primary root-bending experiment (Figure 5I). Taken together, these results suggest that *CLE16/17* promote the transition of LRP from stage I to II, thereby safeguarding LR emergence.

### *CLE16/17* Promote the Expression of *HAE/HSL2* During LR Emergence

Having established the role of CLE16/17 in LR emergence, we asked whether they are also perceived by the CLV1-ACR4 complex during this process like the situation in stem cell regulation. To address this question, we first analyzed the phenotype of lateral root formation in *clv1-20* and *acr4-2* mutants and found no significant difference in LRE density for *clv1-20*, but a moderate decrease in *acr4-2* as also shown by previous results compared to wild-type (Supplementary Figure 3; De Smet et al., 2008). Moreover, we treated the *clv1-20* and *acr4-2* mutant roots with CLE16p and observed a comparable increase of LR density for both mutants compared to those in wild-type roots (Supplementary Figure 3). These results suggest that CLE16/17 promote LR emergence independently of CLV1-ACR4.

**Figure 6:**
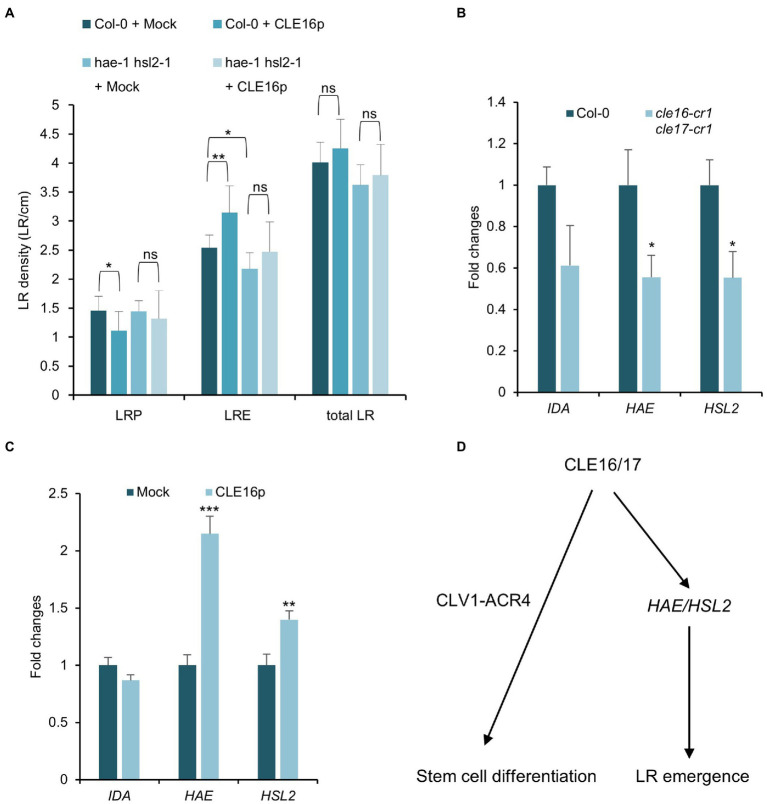
*CLE16*/*17* facilitate LR emergence *via* upregulating the expression of *HAE* and *HSL2*. **(A)** Quantification of LRP, LRE, and total LR density in the seedlings of Col and *hae hsl2* double mutants treated with or without 1 μM CLE16p. One-way ANOVA followed by Dunnett’s multiple comparisons test, ^*^p < 0.05, ^**^p < 0.01, and ns not significant. **(B,C)** Relative expression levels of *IDA*, *HAE*, and *HSL2* in roots detected by qRT-PCR in *cle16 cle17* double mutants compared with wild-type **(B)** and in CLE16p treatment compared with mock treatment **(C)**. PCR signals were normalized to wild-type or mock control. Roots were treated with or without 1 μM CLE16p for 48 h. Error bars represent SD from three biological replicates. Student’s *t*-test, ^*^p < 0.05, ^**^p < 0.01, and ^***^p < 0.001. **(D)** A simplified working model for CLE16/17 regulating root development.

Previous studies have shown that the IDA-HAE/HSL2 signaling pathway plays an essential role in LR emergence (Kumpf et al., 2013; Zhu et al., 2019). Mutations in *IDA* or *HAE/HSL2* cause the delayed developmental progression of LRP and eventually reduced LRE density (Figure 6A; Zhu et al., 2019), which is similar to the observed phenotypes of *cle16 cle17* double mutants. Therefore, we hypothesize that *CLE16/17* act in the same pathway with *IDA-HAE/HSL2* during LR emergence. To test this hypothesis, we treated the *hae-1 hsl2-1* double mutants with CLE16p. In contrast to wild-type, *hae-1 hsl2-1* roots are much less insensitive to the exogenous CLE16p as shown by the unaltered LR density compared to the mock treatment (Figure 6A). These results indicate that the promotion of LR emergence by *CLE16/17* requires *HAE/HSL2*. To further test whether *CLE16/17* function upstream of *IDA-HAE/HSL2*, we performed a gene expression analysis by quantitative reverse transcription PCR (qRT-PCR). In *cle16-cr1 cle17-cr1* double mutants, the mRNA abundance of both *HAE* and *HSL2* is significantly decreased compared to wild-type (Figure 6B). Conversely, in comparison with the mock-treated control, transcripts of *HAE* and *HSL2* are upregulated upon exogenous CLE16p treatment (Figure 6C). Notably, the expression level of *IDA* is not significantly altered in both cases (Figures 6B,C). Taken together, these data indicate that *CLE16/17* promote LR emergence possibly *via* activating *HAE/HSL2* expression.

## Discussion

Peptide-mediated cell-to-cell communication is crucial for plant growth and development. There are 32 *CLE* genes in *Arabidopsis*, and *CLE* members are also found in a variety of species including rice, wheat, and tomato (Goad et al., 2017). However, only a few of them have been functionally characterized (Fletcher, 2020). In this study, we identified two *CLE* members *CLE16/17* in *Arabidopsis*. On the basis of several lines of genetic and molecular evidence, we propose a working model where the CLE16/17 peptides promote distal stem cell differentiation through the CLV1-ACR4 protein kinase complex in roots. On the other hand, CLE16/17 activate the expression of *HAE/HSL2* to facilitate LR emergence (Figure 6D).

### Functional Redundancy Between *CLE* Genes to Promote Stem Cell Differentiation

Mutations in *CLE16* and *CLE17* result in more roots with two layers of starchless CSCs which could be explained in two ways. One explanation is that *CLE16/17* repress CSC division to maintain the homeostasis of the columella root cap. Increased cell division caused by loss-of-function of *CLE16* and *CLE17* leads to more CSCs. However, this explanation is at odds with the fact that synthetic CLE16p treatment is sufficient to alter cell identity from pluripotent CSCs to highly differentiated columella cells. Thus, we favor the other explanation that CLE16/17 promote the exit of CSC daughter cells from stemness during columella differentiation. It is of note that CLE16 has recently been demonstrated to be necessary and sufficient to activate SHR-mediated ACD in cortex endodermal cells and their daughter cells (Crook et al., 2020). Application of CLE16p results in ectopic ACD without compromising cell identity which appears to be distinct from the effect on promoting CSC differentiation by CLE16p observed in our study (Crook et al., 2020). These results indicate that CLE16 functions in a cell context-dependent manner.

Neither *cle16* nor *cle17* single mutants, but *cle16 cle17* double mutants show an increased frequency of roots containing an extra CSC layer as compared to wild-type. This demonstrates a redundant role in stem cell regulation between *CLE16* and *CLE17*. Although *CLE20* is not the focus of our study because of the absence of its expression in the root cap, we cannot exclude the possibility that *CLE20* might compensate when the activities of *CLE16/17* are both depleted. Recently, a study on *CLE* genes conducted in tomatoes has shown that *SlCLE9* is substantially upregulated in the shoot apical meristem of homozygous null mutant *Slclv3*. Enlarged shoot meristem of the *Slclv3* mutant is greatly enhanced when *SlCLE9* is knocked out in the background of *Slclv3* (Rodriguez-Leal et al., 2019). These results demonstrate a transcriptional compensation between *CLE* genes in stem cell regulation. Therefore, we consider the compensation effect for *CLE20* likely.

*cle16 cle17* double mutants show an extra CSC layer, phenocopying the *cle40* mutants (Stahl et al., 2009). Conversely, both CLE40p and CLE16p treatments result in the downregulated *WOX5* expression in the QC and accordingly the differentiated CSCs (Stahl et al., 2009). These phenotypic similarities raise an interesting question regarding the relationship between *CLE40* and *CLE16/17*. One plausible explanation is that they act partially redundantly to regulate stem cell differentiation. Future studies on characterizing a *cle16 cle17 cle40* triple mutant should be helpful to clarify this issue.

### Perception of the CLE16/17 Signaling

Small secreted peptides are mainly recognized by the LRR-RLK membrane proteins, triggering the downstream signaling events to regulate a variety of developmental processes. Here, we propose that CLV1-ACR4 might be the receptor kinase complex that recognizes CLE16/17 ligands during CSC differentiation on the basis of three lines of evidence. First, *CLE16/17* exhibit an overlapping expression pattern with *ACR4* in the columella root cap (Figures 1A,F; Stahl et al., 2009). Second, *cle16 cle17* double mutants exhibit increased CSC numbers similar to the situation in *clv1* or *acr4* mutants (Figures 4F,H; Stahl et al., 2009), suggesting that *CLE16/17* and *CLV1-ACR4* act in the same pathway promoting CSC differentiation. Third, the CSC termination caused by the CLE16p treatment is greatly suppressed in the *clv1* or *acr4* mutant background (Figures 4G,I), suggesting that *CLE16/17* function in stem cell regulation dependently of *CLV1-ACR4*. By contrast, the receptor complex perceiving CLE16/17 might not be CLV1-ACR4 during lateral root formation. Because unlike *CLE16/17*, loss of activity of *CLV1* does not disturb LR emergence (Supplementary Figure 3). In addition, the effect of promoting LR emergence by the CLE16p treatment is not abolished by the loss-of-function mutations in *CLV1* or *ACR4*. We, therefore, conclude that the CLV1-ACR4 receptor complex is not involved in the CLE16/17 signaling pathway during LR emergence.

### *CLE16/17* Facilitate LR Emergence *via* Activating *HAE/HSL2*

Lateral root formation requires a process of cell separation called cell wall remodeling that occurs in the overlaying tissue of the growing LRP (Stoeckle et al., 2018). *HAE* and *HSL2* are mainly expressed in cells overlaying new LRP and the double mutants display defects in pectin degradation which might result in the repression of LR emergence (Kumpf et al., 2013). Given that the similar defects in early LRP development between *cle16 cle17* and *hae hsl2* double mutants, and that loss-of-function of *HAE* and *HSL2* suppresses the promotion of LRE by CLE16p, we conclude that *CLE16/17* and *HAE/HSL2* genetically function in the same pathway during LR emergence. qRT-PCR analysis further reveals their molecular relationship that *CLE16/17* upregulate the expression of *HAE/HSL2* (Figures 6A,B). However, it is still unclear how *CLE16/17* activate *HAE/HSL*2 at the transcriptional level. Given that several *CLE* genes including *CLE6*, *CLE40*, and *CLE41* have been reported to regulate auxin intercellular transport or response (Whitford et al., 2008; Kondo et al., 2014; Pallakies and Simon, 2014) and that both *HAE* and *HSL* are induced by auxin during LR emergence (Kumpf et al., 2013), we speculate that auxin may mediate the regulation of *HAE/HSL2* by *CLE16/17*. Nevertheless, the detailed molecular mechanisms by which *CLE16/17* control stem cell differentiation and LR emergence await further studies.

## Data Availability Statement

The original contributions presented in the study are included in the article/**Supplementary Material**, further inquiries can be directed to the corresponding author.

## Author Contributions

LZ, TI, SS, YZ, and LP conceived and designed this study. LZ, YY, CM, and ML performed the experiments and analyzed the data. LZ, TI, and LP wrote the manuscript. All authors contributed to the article and approved the submitted version.

## Funding

This work was supported by the National Natural Science Foundation of China (31770320 and 31830057) and the start-up fund from Wuhan University.

## Conflict of Interest

The authors declare that the research was conducted in the absence of any commercial or financial relationships that could be construed as a potential conflict of interest.

## Publisher’s Note

All claims expressed in this article are solely those of the authors and do not necessarily represent those of their affiliated organizations, or those of the publisher, the editors and the reviewers. Any product that may be evaluated in this article, or claim that may be made by its manufacturer, is not guaranteed or endorsed by the publisher.

## Supplementary Material

Supplementary Figure 1 Expression patterns of CLEs identified by scRNA-seq. **(A)** No GFP signal detected for pCLE20:3xnlsGFP in primary root tips at 5 dpg. **(B)** Color-coded root cell clusters identified by scRNA-seq. **(C–E)** Feature plots of CLE16 **(C)**, CLE17 **(D)**, and CLE20 **(E)** expression in root cell types corresponding to **(B)**. Expression data of all three CLE genes were obtained from the scRNA-seq database online (https://bioit3.irc.ugent.be/plant-sc-atlas/).

Supplementary Figure 2 CLE16p treatment inhibits root growth. **(A)** Quantification of root length of the cle mutants at 5 dpg and wild-type treated with CLE16p or CLE5p. **(B)** Confocal images of the root tips of the indicated genotypes and the wild-type roots treated with 1 μM CLE16p. Black arrowheads indicate the junction between meristematic and elongation zones. **(C)** Quantification of root meristem size of the genotypes shown in **(B)**. Roots were grown on agar medium supplemented with or without 1 μM CLE16p or CLE5p for 5 days. Error bars represent SD. Student’s *t*-test, ^*^*p* < 0.05, ^**^*p* < 0.01, and ^***^*p* < 0.001.

Supplementary Figure 3 CLE16/17 peptides do not act through CLV1-ACR4 during LR emergence. Quantification of LRE density of wild-type, clv1-20 and acr4-2 at 8 dpg treated with or without 1 μM CLE16p for 2 days. *n* = 30 for each genotype. Error bars represent SD. One-way ANOVA followed by Dunnett’s multiple comparisons test, ^*^*p* < 0.05, ^**^*p* < 0.01, and ^***^*p* < 0.001.
