# Supplementary figures and images for: Control of Root Stem Cell Differentiation and Lateral Root Emergence by CLE16/17 Peptides in Arabidopsis
Source: Front Plant Sci. 2022 Apr 18;13:869888. doi: 10.3389/fpls.2022.869888 (PMC9062579; doi:10.3389/fpls.2022.869888)

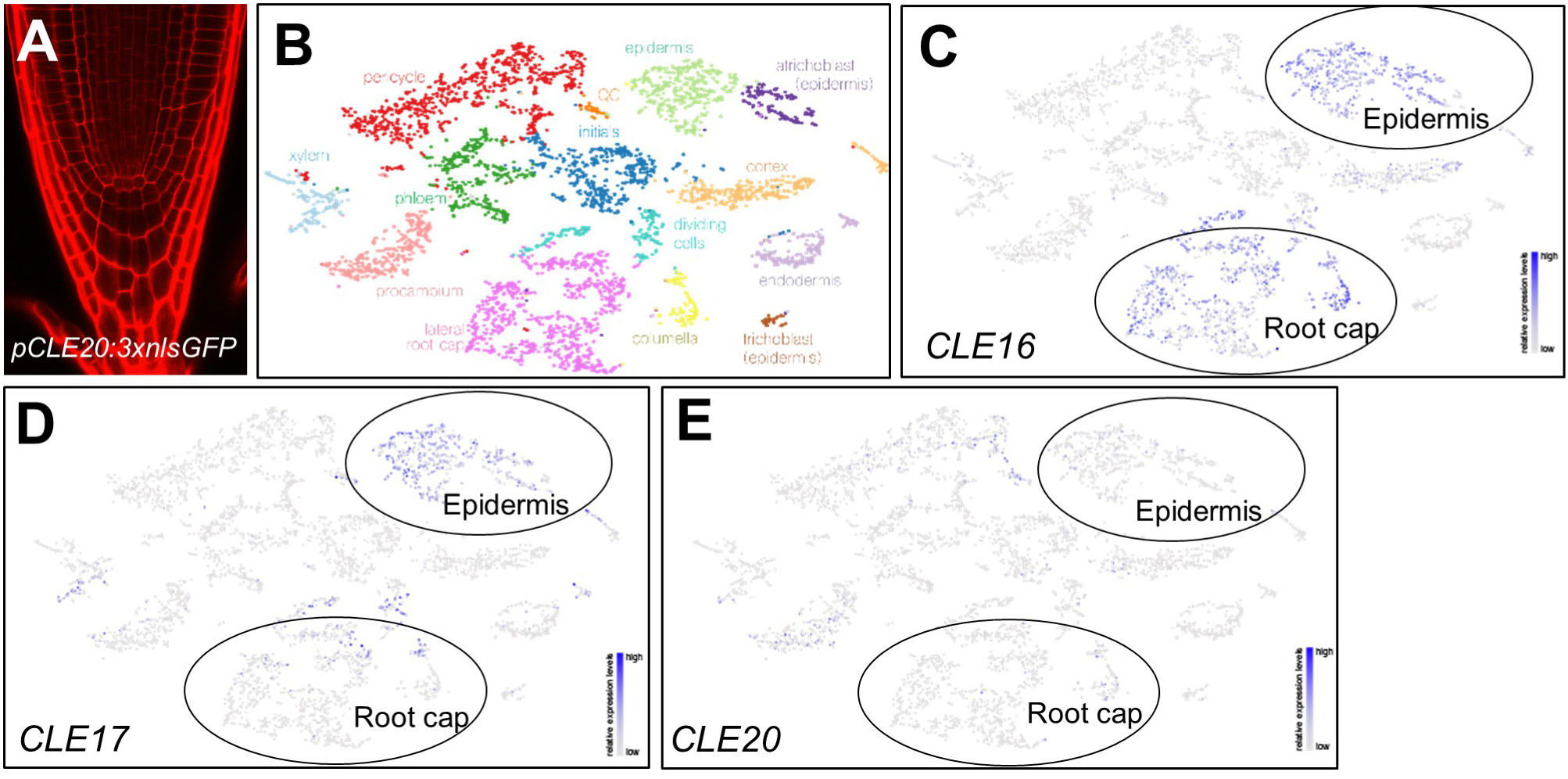

Supplement: Supplementary Figure 1 — Expression patterns of CLEs identified by scRNA-seq. (A) No GFP signal detected for pCLE20:3xnlsGFP in primary root tips at 5 dpg. (B) Color-coded root cell clusters identified by scRNA-seq. (C–E) Feature plots of CLE16 (C), CLE17 (D), and CLE20 (E) expression in root cell types corresponding to (B). Expression data of all three CLE genes were obtained from the scRNA-seq database online (https://bioit3.irc.ugent.be/plant-sc-atlas/). [file Image_1.JPEG]

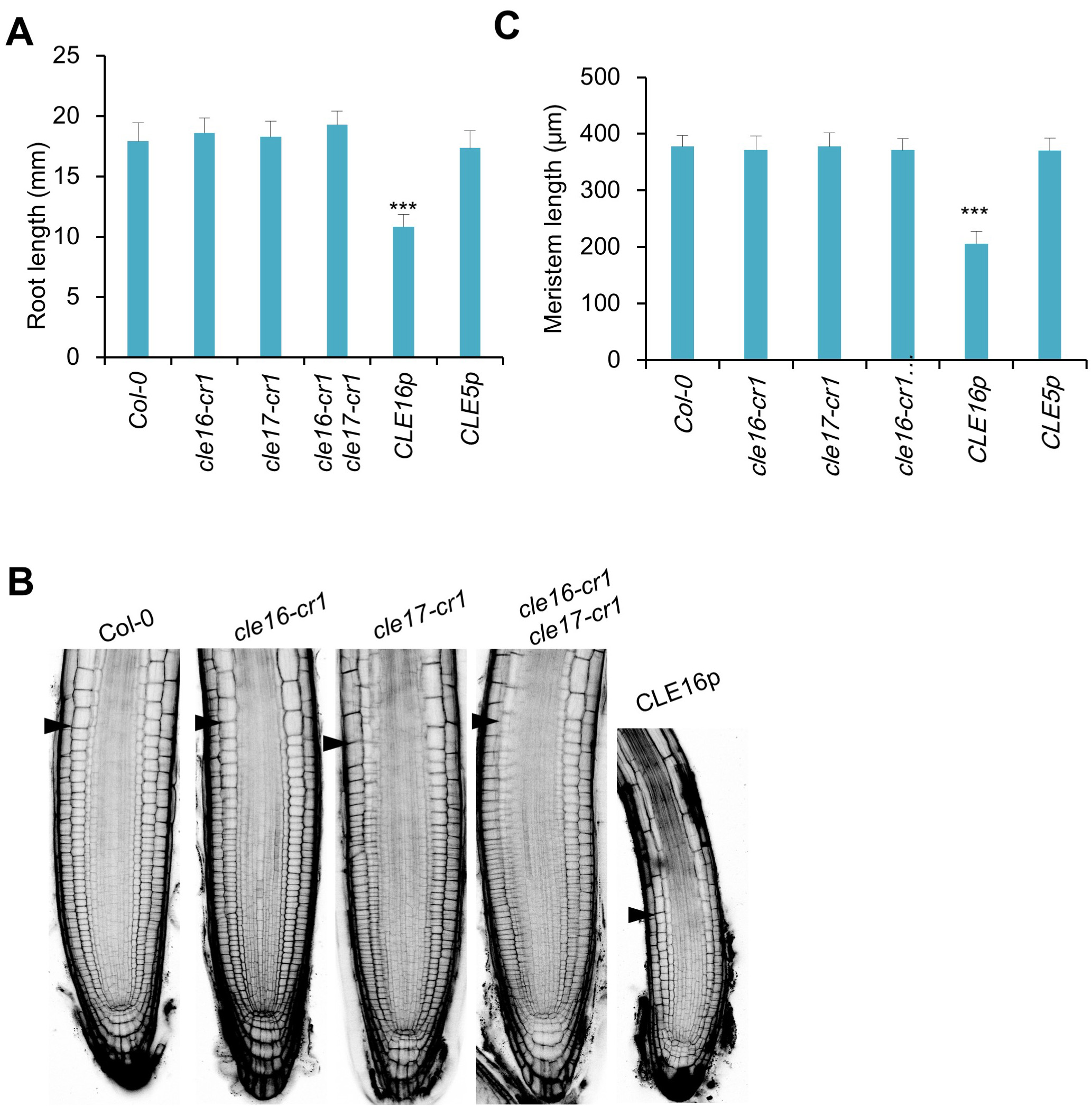

Supplement: Supplementary Figure 2 — CLE16p treatment inhibits root growth. (A) Quantification of root length of the cle mutants at 5 dpg and wild-type treated with CLE16p or CLE5p. (B) Confocal images of the root tips of the indicated genotypes and the wild-type roots treated with 1 μM CLE16p. Black arrowheads indicate the junction between meristematic and elongation zones. (C) Quantification of root meristem size of the genotypes shown in (B). Roots were grown on agar medium supplemented with or without 1 μM CLE16p or CLE5p for 5 days. Error bars represent SD. Student’s t-test, *p < 0.05, **p < 0.01, and ***p < 0.001. [file Image_2.JPEG]

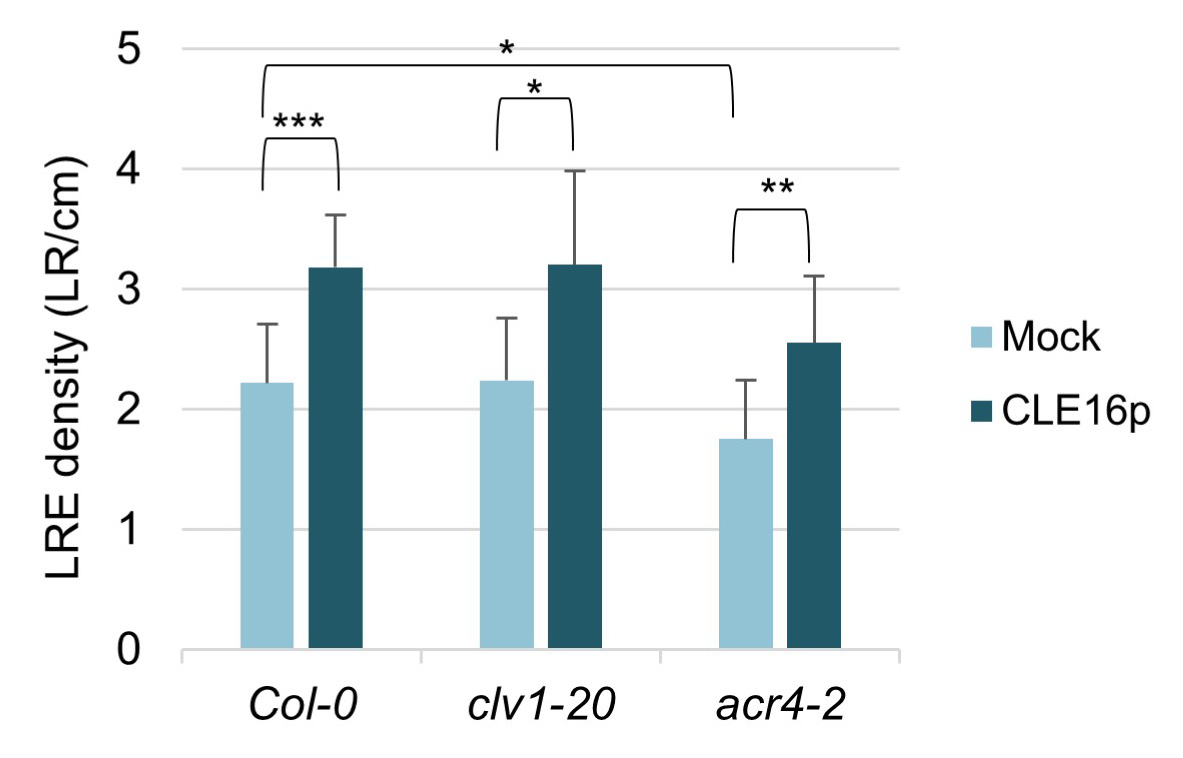

Supplement: Supplementary Figure 3 — CLE16/17 peptides do not act through CLV1-ACR4 during LR emergence. Quantification of LRE density of wild-type, clv1-20 and acr4-2 at 8 dpg treated with or without 1 μM CLE16p for 2 days. n = 30 for each genotype. Error bars represent SD. One-way ANOVA followed by Dunnett’s multiple comparisons test, *p < 0.05, **p < 0.01, and ***p < 0.001. [file Image_3.JPEG]
