# Supplementary material for: Control of Root Stem Cell Differentiation and Lateral Root Emergence by CLE16/17 Peptides in Arabidopsis
Source: Front Plant Sci. 2022 Apr 18;13:869888. doi: 10.3389/fpls.2022.869888 (PMC9062579; doi:10.3389/fpls.2022.869888)
Supplement: Supplementary file 4 [file Table_1.DOC]

**Table 1 Primers used in this study**

| **Name** | **Sequences（5’-3’）** | **Purpose** |
| --- | --- | --- |
| CLE16-F | ATGGAAGCTTGTTCCAGAAAAAGAAG  AAGGAGACGAGC |  |
| CLE16-R | TCAGTTGTGAAGAGGATTTGGACCTGT  GTGAAC | Cloning for  complementation |
| CLE17-F | GGAACAAGGTTTTGTGAGATGACTCACGTGTTGG |  |
| CLE17-R | TTAGTTGTGGAGAGGATTGGGACCCGTGTG | Cloning for  complementation |
| pCLE16-5'-F | CGAGCTCCTTCACTTACTGGTGCTCTT |  |
| pCLE16-5'-R | TCCCCCGGGTTCTGGAACAAGCTTCCA | Cloning for complementation  and transcriptional reporter |
| pCLE16-3'-F | AACTGCAGCCTCTTCACAACTGAAACAC |  |
| pCLE16-3'-R | CCCAAGCTTCAGGGACAAGTATCGAAG | Cloning for complementation  and transcriptional reporter |
| pCLE17-5'-F | CGAGCTCGTAAGGAATTTAGGAGATGA |  |
| pCLE17-5'-R | TCCCCCGGGCTCACAAAACCTTGTTCCG | Cloning for complementation  and transcriptional reporter |
| pCLE17-3'-F | AACTGCAGCCTCTCCACAACTAACT |  |
| pCLE17-3'-R | CCCAAGCTTCCCTGATCACTCACTCAA | Cloning for complementation  and transcriptional reporter |
| pCLE20-5'-F | GGGGTACCAACATGCGAAGAAGCTCGAG |  |
| pCLE20-5'-R | CGGGATCCACGAGGACGACTCGGATTC | Cloning for transcriptional reporter |
| pCLE20-3'-F | ACGCGTCGACGGAAAGAAGAAAATCAGA |  |
| pCLE20-3'-R | CCCAAGCTTTTTGACTTTTAACCCGAG | Cloning for transcriptional reporter |
| cle16-cr1-F | CAGAGATAGATACAAACTC |  |
| cle16-cr1-R | TCAGTTGTGAAGAGGATTTGG | Genotyping for *cle16-cr1* |
| cle17-cr1-F | CGGAACAAGGTTTTGTGAG |  |
| cle17-cr1-R | GTTAGTTGTGGAGAGGATTG | Genotyping for *cle17-cr1* |
| IDA-F | GGTTACTTACCTAAAGGCGTTCC |  |
| IDA-R | AACAAAAGAGTTGTGTCTCTTAGAAGG | qPCR for *IDA* |
| HAE-F | CCCGACAACTTGAATCTGTTAAA |  |
| HAE-R | TCTCCGGTAAAGGACCTT | qPCR for *HAE* |
| HSL2-F | AAAACCGAAACGGACCAAC |  |
| HSL2-R | TTGCGGGTATATGTCTTCCTC | qPCR for *HSL2* |
